# The Treatment of Consumption

**Published:** 1882-07-20

**Authors:** M. L. James

**Affiliations:** Professor of Materia Medica and Therapeutics in the Medical College of Virginia Richmond, Va.


### TREATMENT OF CONSUMPTION INDICATED BY THE DISCOVERIES OF KOCH AND OTHERS OF ITS PARASITIC ORIGIN.

BY M. L. JAMES, M. D.,

**Professor of Materia Medica and Therapeutics in the Medical College of Virginia Richmond, Va.**

The ranking of consumption among the zymotic diseases, which seems at least now to be proper, the next question is the remedy for it. Perhaps in time this may be secured, in the form of prophylaxis, by innoculating with germs of the disease of mitigated virulence, as in the case of vaccination in small-pox, and Pasteur's method for splenic fever. Pending its discussion, no other subject of more pressing importance in medicine or the political or social economy of our race can engage the attention of the profession than measures at once to lessen the destructive force of this, the deadliest disease of all that "flesh is heir to."

For many years the natural history of tuberculosis, and the therapeutic results which I have observed from the use of remedies of germicidal power, has excited in my mind suspicions of its zymotic nature. These suspicions have gradually grown upon me into strong and positive convictions, though I have not had the opportunity of detecting parasitic growth by microscopic observation. Four years ago, in a paper entitled "Consumption: the Question of its Curability and Treatment," which I had the honor to read before the Medical Society of Virginia, and which was published in its *Transactions*, I called attention to the prominence of the febrile phenomena of phthisis and to the salutary results of remedies addressed especially to that feature of the disease, citing several cases of distinctly defined phthisis which, chiefly under the use of such remedies, had secured periods of exemption from the disease ranging from one to nine years, and seemed in all respects to be cured.

Subsequent observations have so strengthened my convictions, that for the last three years my treatment of consumption has been almost exclusively confined to agents belonging to the antizymotic class, and in view of the curative results I had secured, I had felt authorized to declare, as I did, to our classes in the Medical College of Virginia, my belief that consumption was essentially a zymotic disease, and that its proper therapeutics is founded in such recognition.

Within the past thirteen years I have kept the record of twenty-three cases of phthisis of well marked diagnosis occurring in my private practice, which have been relieved of all constitutional symptoms and physical signs for periods ranging from eighteen months to now thirteen years, most of the subjects exhibiting as high a present degree of physical health and strength as average persons in the community. All the patients whose cases ended thus favorably fell under my charge in the first stage of the disease, and most of them with the earliest positive expressions, except two. In these two cases a cavity of the size of a walnut had occurred to one, and two smaller ones had occurred to the other.

Besides the twenty-three cases, one other seemed to be well for a period of nine years, who had a subsequent attack and died. It would seem to me, however, as illogical to conclude that this case was not cured, with all traces of the disease absent for so long a time, as to conclude that a person cured of malarial fever, who had at such an interval had a subsequent attack of malarial fever, was not cured in the first instance.

It is, perhaps, proper for me to say that I have kept the record of sixty-two other cases, treated at various stages of the disease, fifty-one of which have died, and the issue of eleven others is yet undecided, they still being under treatment.

My earlier successful cases were treated chiefly by quinine, carbolic acid in form of inhalation, glycerine and alcohol; by rest, pure air, timely exercise, and suitable food, all of which measures are, under varying circumstances, to a greater or less extent, anti-zymotic.

In addition to these remedies, in different conditions, I now also use salicylic acid, salacin, sulphur and its compounds, especially the hyposulphites, arsenic, and paintings with iodine, some of which is absorbed, and besides its counter irritant effect, exerts antiseptic power. I regulate, as far as practical, with mathematical precision, the external temperature—never allowing it to be high enough to promote fever or debility, or low enough to produce congestions or colds. Further experience will enable us to determine which of the anti-zymotic remedies is most efficient, just as we now know that quinine is *the* specific for malarial fever, and sulphur for itch; or if the researches in pathology shall establish different forms of tuberculosis, then which remedy is best adapted to individual pathological types.

If asked to explain how these remedies act, I will answer distinctly, what has been implied already, probably by destroying the organic parasite which produces the disease, just as we know that sulphur does in a case of scabies, and its compounds do in scald-head and fermentative indigestion, and quinine probably does in malarial fevers.

Pressing engagements prevent my giving at this time the details of the treatment I employ. I will say this, however, that ordinarily, as I have before intimated, I build my hopes of success on *early treatment.* It has, at least, not as yet been my good fortune to successfully grapple with the difficulty of this dangerous zymotic poisoning, complicated with structural disorganization of vital organs. And for the reason that I believe that this cannot be done, and the earlier treatment is commenced, the greater the chances of success, I know of no more important subject in practical medicine than the early diagnosis of tuberculosis. Happily, with the present resources of our art, this may, in almost all instances, be made demonstrative. But to compass it will, in many instances, require a ready familiarity with the significance of the whole range of constitutional symptoms and physical signs. The accurate recognition of fever, its degree and variations, is especially important, This can of course only be done by the use of the thermometer. In taking charge of a case of tuberculosis, I always see that the patient or his attendants are provided with a tested thermometer, instructed in the proper use of it, and required to keep a record of the temperature at all periods of the twenty-four hours, impressing the importance of care by telling them that the success of the management of the case will depend upon the ability to control the fever. My experience justifies me in saying that in a considerable proportion of cases this may be done, and the morbific agency, whatever it may be finally settled upon as consisting in, may be destroyed.

The Materia Medica, in its more limited range in the drug-stores, and its wider range in the universe of matter and mind, must be boldly but practically handled, and the patient watched with ceaseless vigilance. I attend my cases of tuberculosis, at least the more acute forms of it, almost as closely as I do my cases of typhoid fever, ready to combat at once all hurtful changes.

To patients favorably situated, in the earliest stages, where the digestive organs will tolerate for a considerable length of time maximum doses of the anti-zymotic agents mentioned, I believe that a larger number of favorable final issues may be expected than I have yet been able to report. My proportion of cures in recent years much exceed those formerly, as I understand better the plan of treatment, and employ it with more courage and decision.

Unhappily for the doctor, he is usually not called in the earliest stages of tuberculosis, or if so, the digestive organs of the patient will not tolerate the remedies in sufficient quantities and for a sufficient length of time to enable him to destroy the morbific cause. In such cases he must exercise a practical ingenuity by introdcing them in other than the usual channels. Inhalations now, then enemata, then hypodermic injections, epidermic methods, or otherwise.

It is proper for me to remark, that while my chief reliance is placed upon remedies of the anti-zymotic class, in the changing conditions which are liable to occur in this disease I do not ignore entirely the remedies that have been more usually employed. And I wish here to reiterate my abiding confidence in the value of a succession of blisters.

And I will take occasion to say that ordinarily it is best that the friends of the patient and the patient himself should be frankly informed as to the nature of his malady. A wise discretion should of course be employed here as to the subjects, the time, and the manner of making this announcement, but I do not remember ever seeing any but ultimate good results come from such a communication properly made. It will produce some shock and momentary depression with the patient, but that will be all. For a patient to be permanently despondent, is almost pathognomonic of the absence of phthisis. While if properly informed with such assurances as we may reasonably and truly give him, his courage, his hopes and his exertions will usually exceed even those of his doctor. Hopefulness with a consumptive amounts to a monomania, but it is a monomania which his physician may utilize.

If time allowed I should be glad to refer to other facts and considerations in connection with this important subject.—*Virginia Med. Monthly.*

